# Experimental investigation of sharp-tip microwire-based brain electrode buckling during implantation through dura and pia mater

**DOI:** 10.3389/fnins.2026.1774302

**Published:** 2026-04-28

**Authors:** Dongyang Yi, Joshua Thorson, Njinang Kwankam, Lei Chen

**Affiliations:** 1 Department of Mechanical and Industrial Engineering, University of Massachusetts Lowell, Lowell, MA, United States; 2 Department of Mechanical Engineering, Louisiana Tech University, Ruston, LA, United States

**Keywords:** buckling, dura mater, effective length factor, microwire brain electrode, pia mater

## Abstract

**Introduction:**

Wide use of miniaturized and flexible microwire electrodes faces challenges of wire buckling against the brain membrane layers. The field lacks quantitative understanding of such buckling phenomena, especially on the effective length factor, which is required to determine the wire’s critical buckling load.

**Methods:**

This study presents an experimental investigation into the buckling behavior of tungsten microwire electrodes during implantation through dura and pia mater layers using a validated multilayer brain-mimicking phantom. Microwires with three diameters (25.4, 50.8, and 76.2 µm) and different tip geometries—including blunt, beveled, and electrochemically (conical) sharpened profiles—were evaluated under controlled axial insertion. Critical buckling length, insertion outcomes (buckled/penetrated), and rupture/buckling force were quantified across the experimental dataset. Buckling behavior was analyzed using the Euler column framework with experimentally estimated effective length factors (*Kˆ*) to represent each unique membrane-wire tip boundary interaction.

**Results:**

Results indicated that wire diameter strongly influences buckling resistance, with larger diameters yielding quartic (fourth order) higher critical buckling load of the electrode, whereas the corresponding membrane rupture force only increases linearly with the diameter. But smaller microwires tend to anchor better against the brain membrane, generating a more stable wire-membrane interface closer to the ideal pin end condition. Tip geometry also significantly affected rupture force and insertion stability; conical tips dramatically reduced the membrane rupture force with less variance. In general, tip sharpening choice for small microwires should focus on optimizing tips anchoring mechanism and minimizing rupture force uncertainty introduced by tip asymmetry while thick microwires mainly benefit from membrane rupture force reduction. For theoretical prediction of a microwire electrode’s critical buckling load based on Euler’s buckling equation, unlike conventional fixed-pinned assumption (*K* = 0.7), experimentally measured effective length factors ranged from approximately 0.72 – 0.82.

**Discussion:**

Designing with ≈ 0.8 provides a conservative estimate that may reduce the risk of buckling under membrane penetration conditions compared to the commonly assumed fixed-pinned value of 0.7. These findings provide quantitative design guidance for optimizing microwire geometry and offer a validated benchtop framework for predicting buckling-limited insertion performance in neural interface applications.

## 1 Introduction

Implantable microelectrode arrays (MEAs) are the most commonly used technology to detect high-resolution single-neuron action potentials in the brain, which is critical for the fundamental understanding of the brain’s functioning mechanism (Im and Seo, 2016; Yi et al., 2022b; Bargmann et al., 2014; Yi et al., 2024b). Despite substantial recent advances in silicon- and flexible-substrate planar probes as high-density brain–machine interfaces (Chung et al., 2019; Frank et al., 2019; Jun et al., 2017; Melin et al., 2024; Viventi et al., 2011), conventional microwire-based MEAs remain popular and widely used due to their low cost, ease of customization, and accessibility through in-house benchtop fabrication steps (Dong et al., 2020; Guitchounts et al., 2013; Yi et al., 2022a; Chen and Kucernak, 2002; Chen et al., 2021). Recent neuroscience trends toward large-scale chronic *in vivo* recording and stimulation experiments require the use of ultra-fine and flexible microwire electrodes to be implanted to minimize inflammatory responses and mechanical trauma (Bimbard et al., 2023; Barrese et al., 2013; Thielen and Meng, 2021; Chen et al., 2019; Won et al., 2020; Zambrano-Intriago et al., 2021).

Flexible neural electrodes closely match the mechanical properties of brain tissue, which helps reduce chronic inflammatory responses and makes them well-suited for long-term implantation (Viventi et al., 2011; Felix et al., 2013). However, the low bending stiffness of flexible electrodes also makes them more prone to buckling during insertion. Conversely, rigid probes offer greater mechanical robustness, enabling reliable penetration but may increase tissue strain and long-term inflammatory response. Consequently, electrode selection must be tailored to the intended application, considering factors such as recording duration, implantation depth, and spatial resolution needed (You et al., 2024; Zhang et al., 2021). For microwire-based brain electrodes, beyond mitigating tissue response, scaling down the wire diameter also enables higher electrode packing density within a constrained brain volume. This consideration is particularly important because conventional microwires typically provide a single recording site at the tip, whereas silicon-based probes can integrate multiple recording contacts along the shank (Frank et al., 2019; Marshall et al., 2016). Therefore, reducing microwire size supports large-scale neural population recording while preserving the compliance advantages desirable for chronic implantation (Yi et al., 2024b). However, decreasing diameter simultaneously reduces bending stiffness, thereby increasing susceptibility to buckling during membrane penetration (Barrese et al., 2013). Understanding the mechanics governing insertion stability is thus critical for enabling flexible electrode designs that can achieve both minimal tissue disruption and reliable implantation. Implantation of such miniaturized microwire electrodes faces a major surgical barrier: the buckling of the microwire electrodes against the brain membrane layers (Yi et al., 2022b; Thielen and Meng, 2021; Tian and He, 2005). For example, in rodent recording studies, the pia mater has exhibited significant difficulties with small microwires, such as 12 μm-diameter tungsten and 7 μm-carbon fibers (Dong et al., 2020; Patel et al., 2015; Patel et al., 2020). The thin, tough dura mater is usually considered infeasible and thus requires surgical removal. Such a durotomy process causes additional trauma to the brain and prevents large-scale brain-wide recording experiments, given the surgical challenges for a brain-wide durotomy. Thus, a persistent design challenge arises: the microwire must be sufficiently stiff to penetrate through protective brain membrane layers yet compliant enough to ensure chronic performance (Yi et al., 2022b; Thielen and Meng, 2021). Lack of a thorough understanding of the dura and pia penetration process and buckling prevention mechanism leads the community to use thicker, more damaging electrodes to reach deep brain structures, or to perform hours-long surgeries to create insertion windows without the membranes at targeted locations.

Microwire buckling happens when the insertion force (axial) against the brain membranes (*F*) is larger than the microwire’s critical buckling load (*P_crit_*), which is evaluated by Euler’s column buckling equation (Cernica, 1966):

$$P_{\text{crit}}=\frac{\pi^{2}\;\;E\;I}{{\left(K\;L\right)}^{2}}$$ (1)

where *E* is the Young’s modulus (determined by microwire material), *I* is the second moment of inertia (determined by the wire diameter), *L* is the unsupported length (dependent on desired insertion depth), and *K* is the effective length factor based on the column boundary conditions. Although unsupported length is associated with the targeted insertion depth during conventional stereotaxic implantations, several engineering strategies have been developed to reduce the effective unsupported span while enabling access to deeper brain regions. These include the use of insertion guides (Yi et al., 2022a; Chen et al., 2019), temporary stiffening coatings (Patel et al., 2015), shuttle-assisted delivery systems (Thielen and Meng, 2021), microdrive-supported implantation (Yi et al., 2024b) and incremental grip-feed-release (Smith et al., 2022; Yi et al., 2024a) approaches that provide lateral support of the electrode during penetration. Such methods highlight that unsupported length is not purely an anatomical constraint but also a controllable design parameter influencing buckling susceptibility. The peak insertion force appears at the membrane layer rupture (*F_rupture_*), before which the buckling is most likely to happen, as further insertion after rupture is against wire-brain friction only with decreasing unsupported length. Thus, to prevent buckling, the key overall strategy is to decrease the membrane rupture force and increase the critical buckling load.

To enhance the microwire electrodes’ membrane-penetration capabilities, the most common practice is to sharpen the microwire tip. Major techniques for manufacturing mental microwires include electrochemical machining, electrical discharge machining, mechanical polishing, as well as fire torch burning and laser cutting for carbon fiber microwires (Dong et al., 2020; Guitchounts et al., 2013). Sharpened wires have been shown to reduce the membrane rupture force in previous studies (Chen et al., 2021) for pia penetration in rodent and zebra finch models. However, to determine the optimal sharp-tip for various wire sizes, the field still lacks a quantitative understanding of how different types of microwire sharp tips affect the membrane rupture force, particularly when the more difficult-to-penetrate dura mater layer is included. Prior studies have investigated the mechanics of neural probe insertion across multiple electrode configurations. For example, Obaid et al. (2020b) reported that both microwire diameter and tip geometry strongly influence penetration force and insertion reliability in microscale electrodes, underscoring the importance of geometric optimization for minimizing tissue damage while preventing structural failure. Recent work on electrochemically sharpened microwire electrodes (McNamara et al., 2024) has similarly demonstrated that reducing tip radius can significantly lower membrane rupture force and improve insertion consistency. Beyond microwires, silicon-based Michigan-style probes face analogous mechanical challenges during implantation (Wellman et al., 2018), where sufficient bending stiffness must be maintained to avoid buckling while limiting tissue trauma. Force measurement studies on these probes have provided valuable insight into how probe geometry, insertion conditions, and boundary interactions govern penetration behavior (Yi et al., 2022b; Thielen and Meng, 2021; Perna et al., 2025). Together, these findings highlight that successful neural interface design fundamentally requires balancing penetration force with column stability—a mechanics framework that motivates the present experimental investigation.

**Figure 1:**
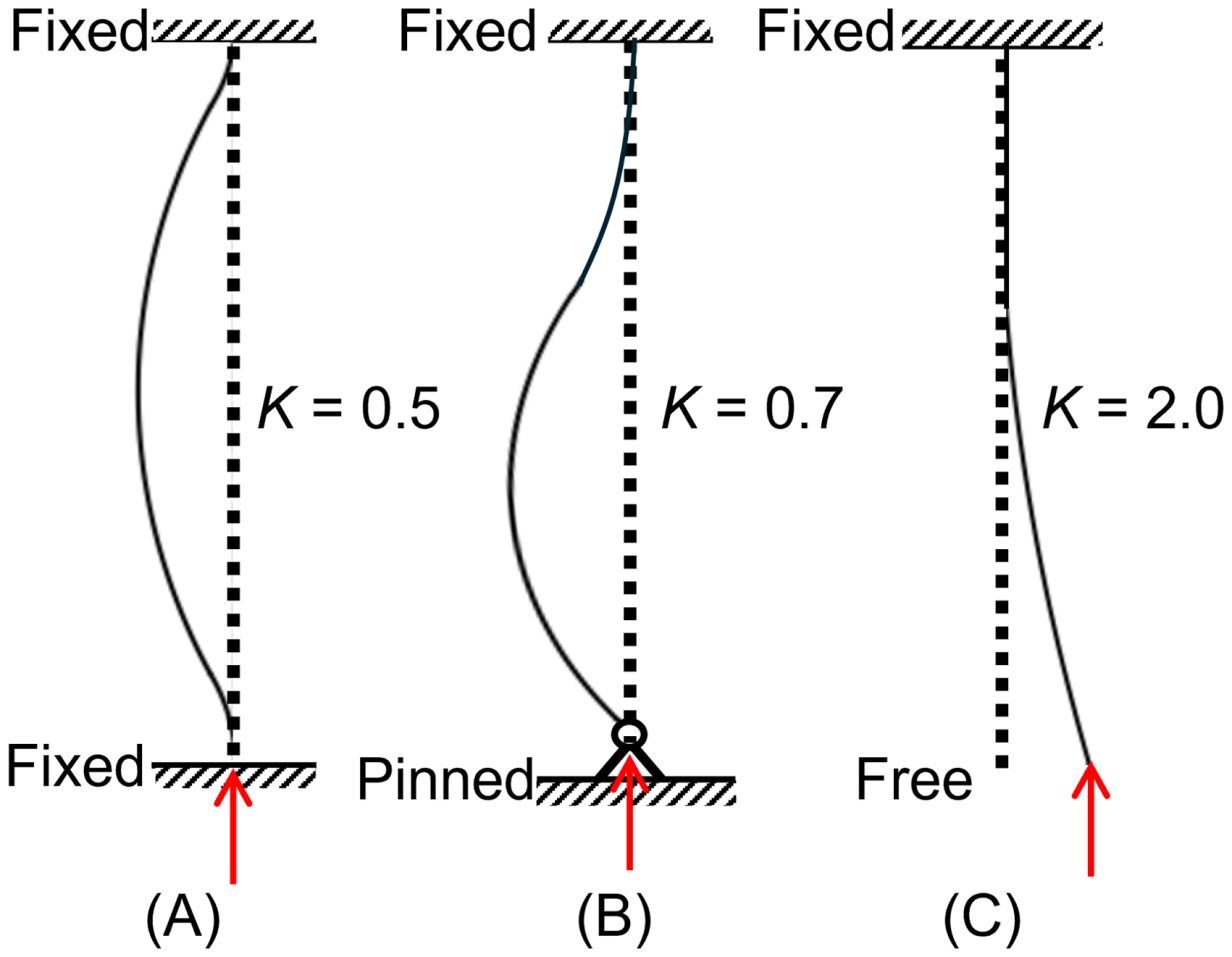
Dependence of the effective length factor *K* value on the wire tip – brain membrane interface boundary conditions: **(A)** *K* = 0.5 for fixed-fixed condition, **(B)** *K* = 0.7 for fixed-pinned condition, and **(C)** *K* = 2.0 for fixed-free condition.

On the critical buckling load side, while the microwire material, size, and targeting area clearly define the *E*, *I*, and *L* in Equation 1, the effective length factor *K* remains largely unknown for brain electrode penetration cases. Given that the top end of the microwires is connected to MEA circuit boards, it is usually considered a fixed condition. However, depending on the wire-brain membrane interface boundary condition, the effective length factor can vary dramatically (Figure 1). For insertions with a custom guide and full support at the brain top surface toward a fixed end condition, the *K* value may be close to 0.5 (Figure 1A). For conventional stereotaxic insertions, it is usually assumed to be a pinned end between the wire tip and the brain (Marshall et al., 2016; Jeanpierre et al., 2025), yielding a *K* value of 0.7. Such an assumption is usually questionable, as the real boundary is not an ideal pinned connection; the potential for wire slipping on the soft brain surface increases the chances of buckling (increased *K* toward larger values, with the extreme being a free wire end for *K* = 2.0). According to Euler’s column buckling equation (Equation 1), the critical buckling load exhibits an inverse quadratic dependence on the effective length factor, indicating that even modest increases in *K* can substantially reduce the electrode’s buckling resistance. Accurate estimation of *K* is therefore essential for reliable prediction of allowable unsupported length during neural electrode implantation. Furthermore, different blunt and sharp microwire tips affect this critical length factor. Their impacts will also influence the choice of optimal sharp-tip geometry for microwire electrodes.

To gain a quantitative understanding of microwire buckling against the dura and pia mater during brain insertions, this study experimentally investigated the impact of various microwire sizes and tip geometries on both the membrane rupture force and microwire effective length factor (thereby affecting the wire’s critical buckling load). Combined effects of the two aspects were evaluated by experimentally measured critical buckling length, the longest wire that could be inserted without buckling under each wire condition. Experiments were carried out by inserting various wires into a multi-layer brain-mimicking phantom, whose rupture and dimpling properties were calibrated and validated using *in vivo* Sprague–Dawley rat brain insertion test data.

## 2 Materials and methods

### 2.1 Preparation of microwires

In this study, bare tungsten microwires (99.95% pure tungsten wire with 411 GPa Young’s modulus by Midwest Tungsten Service, Willowbrook, IL, USA) of three different diameters (25.4, 50.8, and 76.2 μm) were used for experimental investigation. Tungsten, a commonly used brain electrode material, was selected for its high elastic modulus and structural stability, allowing controlled investigation of the microwire buckling mechanism in this study. In our previous study measuring *in vivo* membrane rupture force and dimpling depth (Chen et al., 2021), no statistically significant differences were observed among different microwire metal materials, suggesting that the results obtained with tungsten wires should also apply to other popular metal microwire choices, such as Platinum-Iridium or Stainless Steel. For each microwire size, seven different tips were fabricated, namely: (1) blunt tip, single beveled tip with (2) 15°, (3) 30°, and (4) 60° bevel angle, and conical sharp tips of three different taper angle levels generated by electrochemical machining processes named as (5) High-Voltage Bath (HVB), (6) Low-Voltage Bath (LVB), and (7) Low-Voltage Dip (LVD). Detailed tip preparation process for each tip type is elaborated below. Three diameter levels and seven different tip configurations yielded 21 wire types investigated in this test.

#### 2.1.1 Polishing process for blunt and single-bevel tips

**Figure 2:**
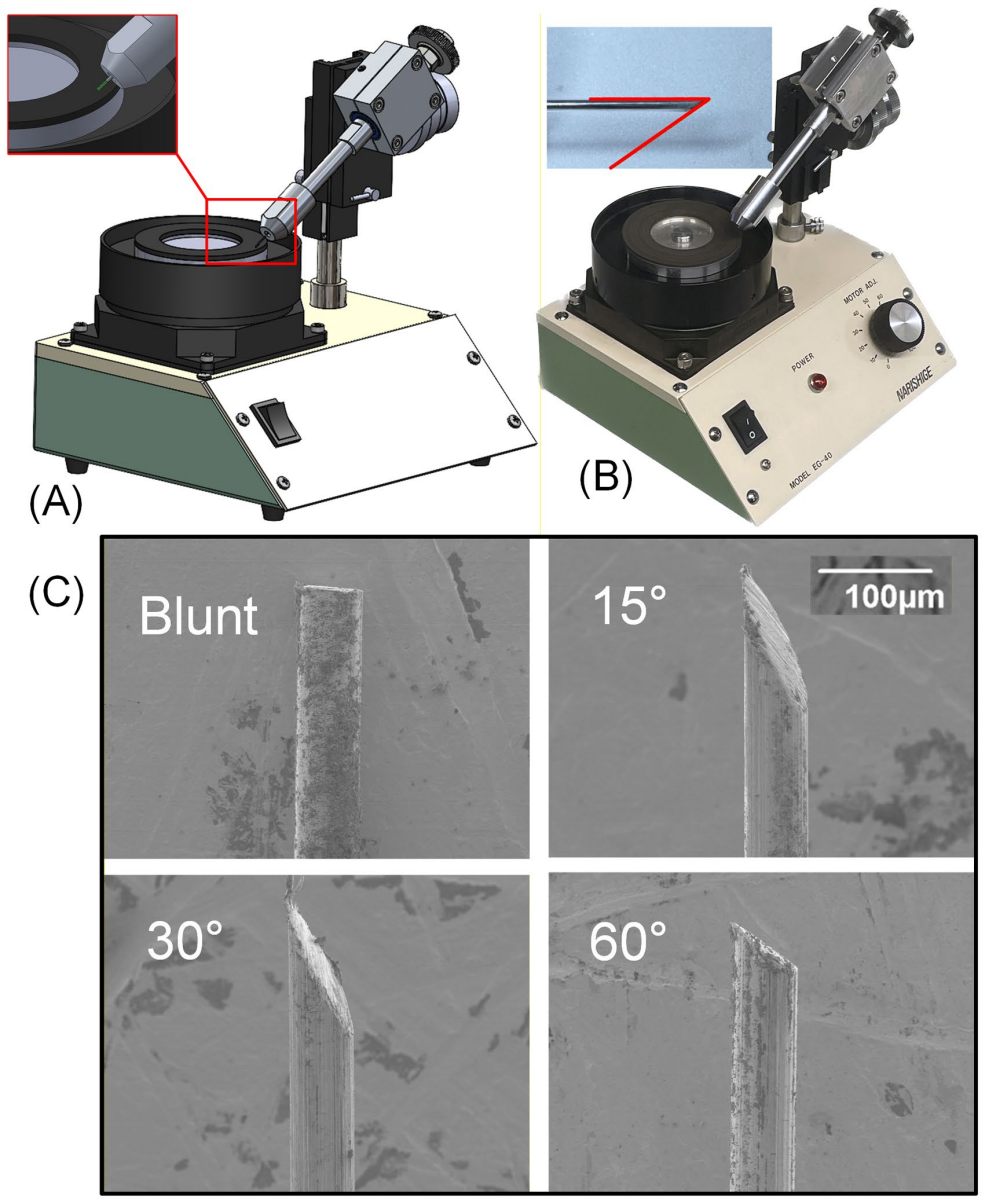
Polishing setup for blunt and single-bevel tip creation: **(A)** diagram and **(B)** picture of a modified micropipette grinder with collet to hold microwires in glass pipette at custom angle for beveled grinding, and **(C)** example scanning electron microscope (SEM) images of the achieved sharp wire tips at various angles (e.g., blunt, and beveled wires at 15°, 30°, and 60°).

Blunt and single-beveled microwire tips were fabricated using a custom precision polishing setup modified from a commercial micropipette grinder (EG-45 by Narishige Scientific Instrument Lab, Tokyo, Japan) by replacing the micropipette holder with a more rigid and precise metal shaft and a collet, as shown in Figure 2A. Before polishing, microwires were bundled and snugly inserted into borosilicate glass capillary tubes to preserve alignment during grinding. Each bundle underwent a multi-step polishing sequence at different grit levels (240 → 1,200, starting from rough grit and moving toward finer grits) at a controlled rotational speed to produce smooth, uniform polishing surfaces. Blunt-tip wire bundles were held vertically using a custom 3D-printed capillary holder to maintain perpendicular contact with the abrasive surface of the grinding wheel. For beveled geometries, the glass capillary holding the snug wire bundle was clamped by the collet and positioned against the abrasive grinding surface at desired bevel angles (e.g., 15°, 30°, and 60°), as in Figure 2B. Representative scanning electron microscopy (SEM) images of the achieved blunt and beveled wires are shown in Figure 2C.

#### 2.1.2 Electrochemically etched conical sharp tips

**Figure 3:**
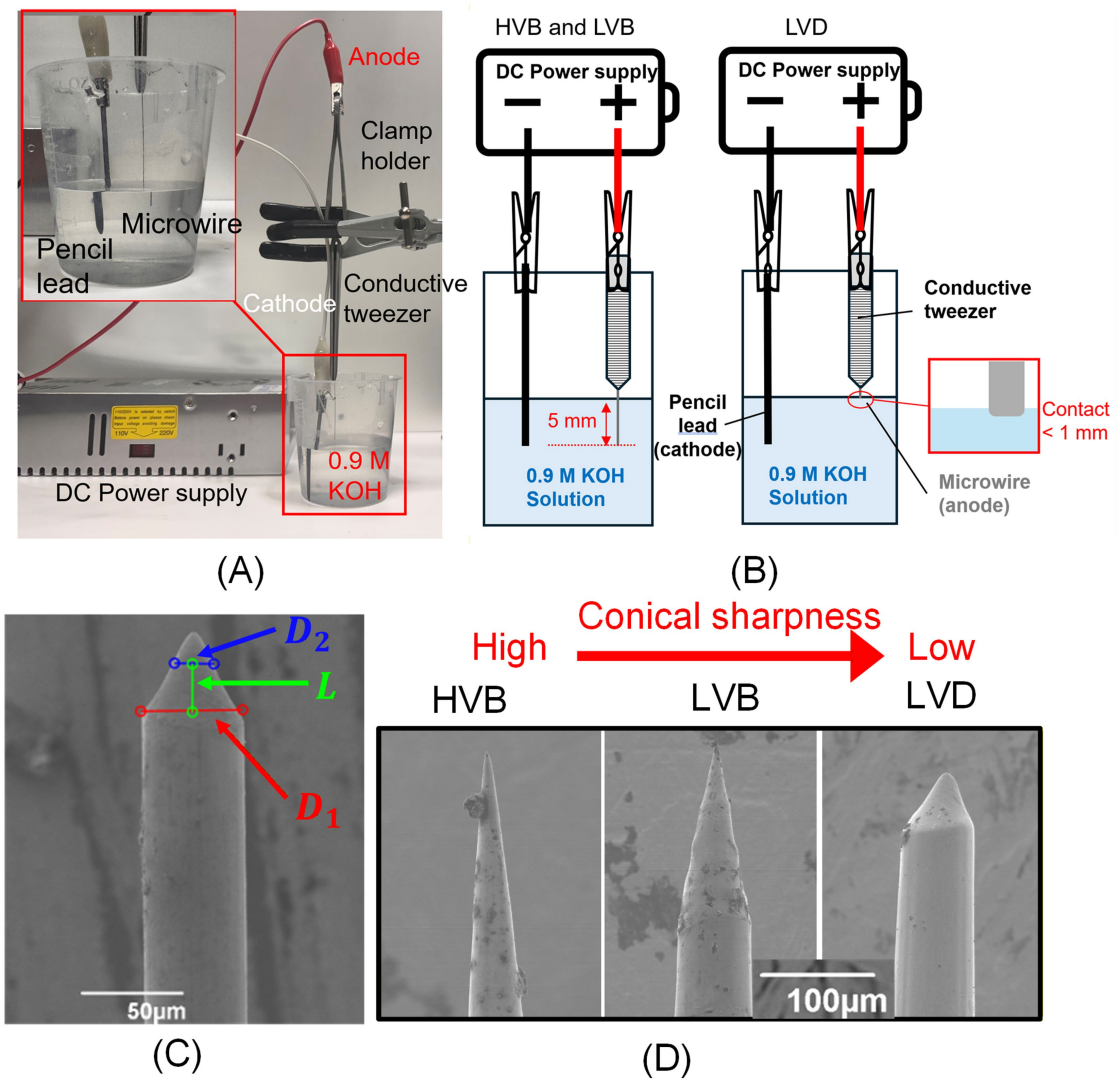
Conical tip sharpening process: **(A)** electrochemical sharpening setup, **(B)** diagram for three sharpening methods, namely (1) High-Voltage Bath (HVB), (2) Low-Voltage Bath (LVB), and (3) Low-Voltage Dip (LVD), **(C)** taper ratio calculation based on SEM imaging, and **(D)** representative SEM images of conical tips from 76.2 μm microwire fabricated under HVB, LVB, and LVD conditions.

Conical sharp tips were created using the electrochemical machining (ECM) process with the setup shown in Figure 3A. The ECM electrolyte in this study was a 0.9 mol/L potassium hydroxide (KOH) solution held in a plastic beaker. A graphite rod (pencil lead) was used as the cathode, and the microwire to be sharpened was the anode. The anode microwire was clamped by a pair of electrically conducting metal tweezers. The back end of the tweezer and the cathode pencil lead were connected to a direct-current power supply using alligator-clip test leads. As shown in Figure 3B, for the High Voltage Bath (HVB) condition, about 5 mm of the microwire was submerged into the electrolyte solution. A 20–32 V (depending on specific wire size) was applied for a prolonged duration (7–10 min) until the microwire breaks off around the electrolyte-air interface. For the Low Voltage Bath (LVB) condition, a procedure similar to HVB was carried out except that the voltage was controlled within a 12–20 V range, and it took 3–5 min for break-off. For the Low Voltage Dip (LVD) condition, the wire tip dipped into the electrolyte with less than 1 mm immersed into the solution, and a 12–20 V DC voltage was applied for 6–20 s (varying based on wire sizes). For each condition, the specific voltage range and etching duration varied with wire diameter and initial length, to ensure uniform taper formation across different wire sizes. These parameters were optimized through extensive iterative experimentation, with several hundred trials performed to establish stable operational windows for each ECM condition. Such varying voltage, immersion depth, and exposure duration resulted in three different conical sharp tips, which were evaluated using SEM images based on the taper ratio. Following etching, all samples were thoroughly rinsed with deionized water, air-dried, and subsequently examined using SEM to verify conical, sharp-tip morphology. Quantitative taper analysis was performed using custom MATLAB® image-processing scripts. As shown in Figure 3C, an SEM side view of the conical sharp-tip was taken, and based on two diameters along the beveled section and their distance, the taper ratio is calculated as (*D_1_*-*D_2_*)/*L*. Representative tips under HVB, LVB, and LVD conditions are shown in Figure 3D. The HVB method yielded the sharpest tip, with a taper ratio of about 0.1–0.2 (corresponding to 6°–12° bevel angle and 12°–24° tip angle). LVB method generated a moderate 0.4–0.6 taper ratio of (22°–31° bevel angle and thus 44°–62° tip angle). LVD created the dullest tip among the three with a 0.9–1.2 taper ratio and 42°–50° bevel angle (84°–100° tip angle). Note that despite being a more efficient sharpening process, as shown in Figure 3D, due to the inherent uncertainty of the sharpening close to the electrolyte-air interface, the LVD tip had a higher likelihood of producing a slightly off-center, asymmetric tip as compared to HVB and LVB ones.

### 2.2 Insertion experiment setup

**Figure 4:**
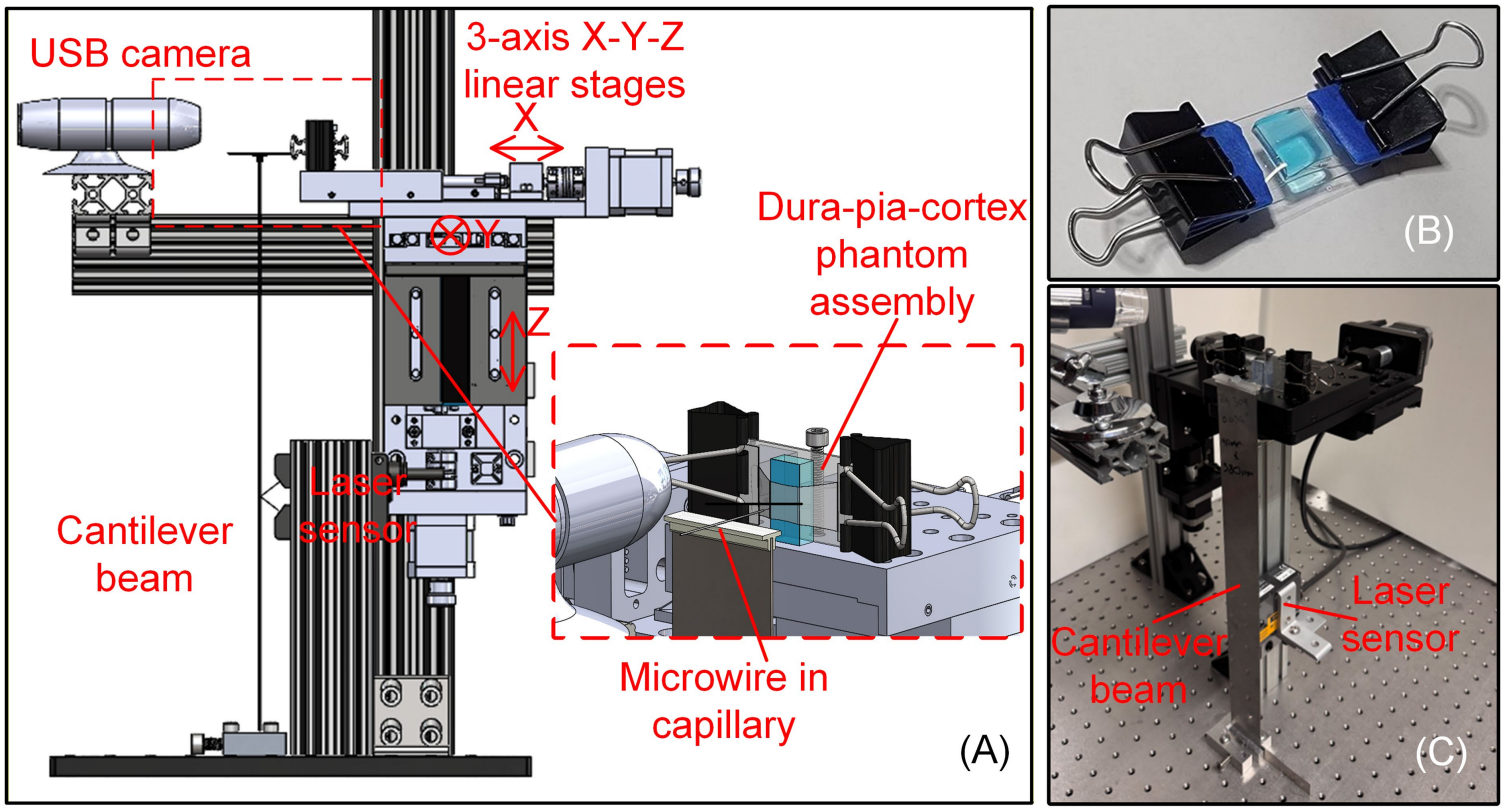
Experimental setup for microwire insertion tests: **(A)** overview of the cantilever beam-based insertion force and dimpling measurement setup, **(B)** picture of an assembled multi-layer dura-pia-cortex-mimicking phantom used in the study, and **(C)** picture of the physical experimental setup.

All 21 types of microwires prepared were used for brain-mimicking phantom insertion test using the setup as shown in Figure 4. The experimental setup was based upon a previously developed and experimentally validated flexible cantilever beam-based insertion force and dimpling depth measurement system (Chen et al., 2021), which was shown feasible for *in vivo* evaluation of both the pia-only and dura-pia penetration processes using microwires (up to 100 μm-diameter tungsten and stainless steel wires tested) or silicon-based probe shanks. As shown in Figure 4A, a stainless-steel cantilever beam (380 mm × 40 mm × 0.91 mm) was fixed vertically using a custom 3D printed cap. The prepared blunt or sharp microwire was placed inside a capillary tube with the desired unsupported length of the wire extending beyond the capillary. The tube with the microwire was then fixed on top of the cantilever beam cap. During each insertion test, a new multi-layer rat brain-mimicking phantom (as in Figure 4B) was used as the target to mitigate the impact of previously cracked dura and pia mater phantom layers. The phantom consisted of three layers to replicate the behavior of different brain layers penetrated by the microwire: (1) a 0.5% weight by volume (w/v) agarose as the cortex/brain tissue layer, (2) a 1.01% agarose (w/v) layer as the pia mater, and (3) a pre-stretched polyvinyl chloride (PVC)–based film as the dura mater. The triple-layer assembly was held in place by a glass slide, two cover slips, and paper clips. Detailed material formulation and fabrication protocols of the brain-mimicking phantom are reported in Yi et al. (2026). The phantom’s mechanical behavior during insertion (membrane rupture force and dimpling depth at rupture) was tuned and validated based on *in vivo* Sprague–Dawley rat brain insertion test data (Chen et al., 2021) conducted on the same cantilever-beam-based force measurement setup as used in this study. Based on the cantilever beam material and configuration, it was calibrated and validated that for a 0.5° tilt of the beam, the force needed at the top of the beam would be 92.5 mN, which well covered the force measurement range in this study (less than 40 mN resulting in less than 0.2° tilt), whose impact on the wire insertion results were treated as negligible.

During each insertion test, the multi-layer phantom was mounted on the motorized linear stage assembly with its center aligned with the mounted microwire. The phantom was advanced toward the microwire at a constant speed of 100 μm/s, the same as the settings from the previous *in vivo* measurement and phantom development work (Chen et al., 2021). Such speed was suggested by Sharp et al. (2009) as a balance between tissue stiffness and electrode adhesion for microwire insertions into rodent brains. A laser displacement sensor (LK-G10 by Keyence Corporation, Osaka, Japan) monitored the real-time deflection of the cantilever beam (Figure 4C). Based on beam deflection at the laser height, real-time insertion force and deflection at the top of the beam could be achieved. In this study, a USB camera was positioned adjacent to the insertion region to continuously record each experiment, enabling synchronized visual confirmation of membrane rupture and lateral deflection associated with buckling. Phantom feeding in each test was stopped when the microwire ruptured the membranes and was inserted into the multi-layer phantom or when the wire buckled. The rupture/buckling point was determined by both insertion force monitoring (sudden beam deflection and a drop in insertion force) and visual confirmation via the camera feed.

### 2.3 Design of experiments and data collection and processing

This study aimed to investigate the impact of microwire diameter and sharp-tip geometry on buckling resistance. Thus, the threshold buckling point was of specific interest. For each one of the aforementioned 21 microwire types (three levels of wire diameter and seven levels of tip geometry), iterative tests were conducted around the critical buckling length value to identify the experimental critical unsupported length to trigger buckling under that wire size and tip shape.

The initial test of each wire type started with an educated guess of the critical unsupported length. After wire preparation and fixation in the capillary tube, the actual unsupported length was always measured under a microscope. If an insertion test yielded a successful penetration, then a longer unsupported length of the same wire would be tested next. If an insertion test results in buckling, the next test for the same wire would use a smaller unsupported length. Such iterative trials were repeated for each wire type until at least three successful penetrations and three buckled tests were conducted, with the insertion force profile recorded by the cantilever-beam-based force-dimpling measurement system. The insertion velocity (100 μm/s) and ambient conditions (22 ± 1 °C) were maintained constant across all tests. In this study, trials for all 21 microwire types yielded 237 insertion tests in total, including 91 successful penetrations and 146 buckled cases.

**Figure 5:**
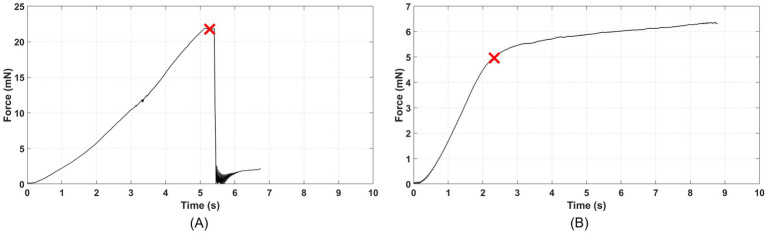
Representative force-time profiles during microwire insertion tests: **(A)** insertion force – time curve of a successful penetration trail with a 50.8 μm diameter bare tungsten (W) microwire with a 60° polished tip and 10.34 mm unsupported length. The cross marker indicates the peak force corresponding to the membrane rupture point, followed by an abrupt force drop associated with penetration through the membrane phantom, and **(B)** insertion force–time curve of a buckled insertion trial with a 50.8 μm diameter bare tungsten (W) microwire with a blunt tip and 20.5 mm unsupported length. The cross marker denotes the maximum force attained at the start of structural instability. Buckling was confirmed by visual observation of lateral deflection via real-time camera recording.

For each successful penetration, the insertion force vs. time curve was plotted, with a representative example shown in Figure 5A. The membrane rupture point was identified on the insertion force curve (usually a sudden drop from peak value) and cross-validated with recorded insertion video clips. Then, the membrane rupture force (*F_rupture_*) was recorded, and the corresponding wire unsupported length (*L*) was measured by a microscope.

For each buckled trial, buckling was identified based on the onset of lateral instability observed in the synchronized video recordings in conjunction with the force response, with a representative example shown in Figure 5B. On the force-insertion time curve, the buckling point usually occurs at the end of the linearly increasing insertion force section. The force corresponding to this instability was used as a practical estimate of the critical buckling load (*P_crit_*), and the microwire had a specific unsupported length (*L*) determined from a microscope measurement. Thus, based on Euler’s column buckling equation as in Equation 1, an experiment-based effective length factor (*Kˆ*) could be calculated for each buckled trial via Equation 2 below:

$$\hat{K}=\frac{\pi }{L}\sqrt{\frac{\left(E\;I\right)}{P_{\text{crit}}}}$$ (2)

### 2.4 Statistical analysis

For each wire type tested, the experimental trials were classified as either buckled or penetrated, with corresponding unsupported length measured for each trial. Such a binary outcome dataset for each type of wire was used for binary logistic regression followed by receiver operating characteristic (ROC) analysis in SPSS (Version 31, IBM, Armonk, NY, USA) to achieve the threshold unsupported length value with the optimal balance point between sensitivity and specificity on the ROC curve (maximum Touden’s index). To ensure the accuracy of the classification model and the threshold unsupported length value, for each wire type, iterative insertion tests were performed with various unsupported lengths to bracket the buckling-penetration transition until (1) at least three successful penetrations were obtained and (2) ROC analysis yielded an area under the ROC being at least 0.8 with less than 0.05 asymptotic significance. The resulting threshold unsupported length value was used as the experimentally determined critical buckling length for the specific combination of wire diameter and tip configuration.

Analysis of variance (ANOVA) was conducted in SPSS (Version 31, IBM, Armonk, NY, USA) to investigate the effects of wire size and tip geometry on buckling resistance (the balance between critical buckling load and rupture force). For the experimental dataset of 146 buckled trials, ANOVA was carried out with the experiment-based effective length factor (*Kˆ*) as the dependent variable, with wire diameter (three levels) and tip-geometry configurations (seven levels) served as the fixed factors. For data from the 91 penetrated trials, another ANOVA was conducted with the membrane rupture force as the dependent variable and the wire diameter and tip geometry as fixed factors. All ANOVAs evaluated the main effects of both factors, with *post hoc* Tukey’s tests used for pairwise comparisons. In this study, a *p* < 0.05 was considered statistically significant.

## 3 Results

### 3.1 Critical buckling length results

**Figure 6:**
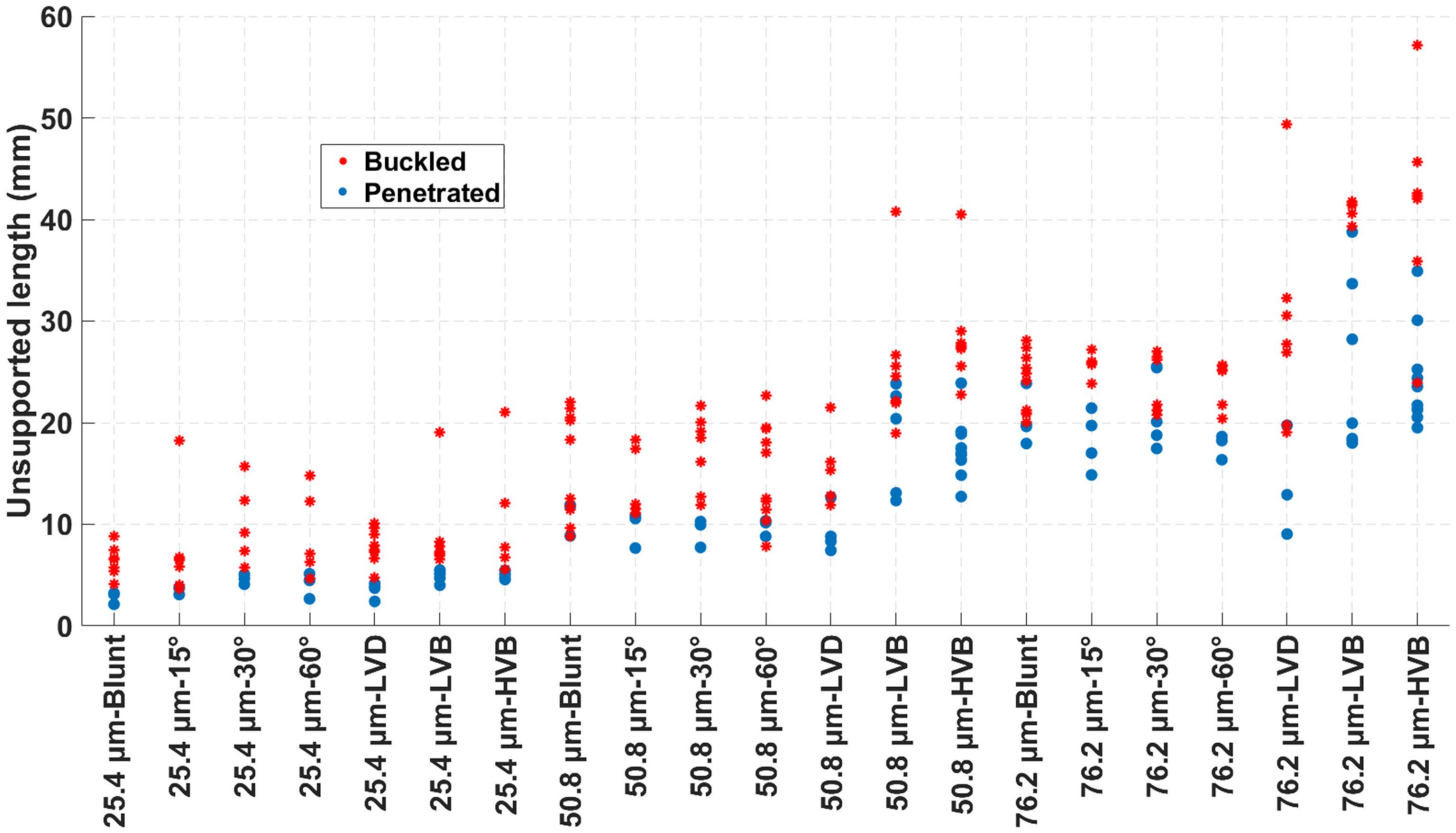
Binary scatter on unsupported length distribution for penetrated and buckled trials across different wire types. Each data point represents an individual insertion trial, grouped by wire size (25.4, 50.8, and 76.2 μm) and tip geometry (Blunt, 15°, 30°, 60°, LVD, LVB, and HVB). Blue-filled circles denote penetrated insertions (successful rupture without buckling), while red asterisks denote failed insertions ending up buckling.

Binary results of all 237 insertion trials are summarized in Figure 6 with the color-coded binary outcome (buckled or penetrated), plotted at the corresponding actual wire unsupported length for each trial. All outcomes are categorized by 21 different wire sizes and tip-geometry combinations.

**Figure 7:**
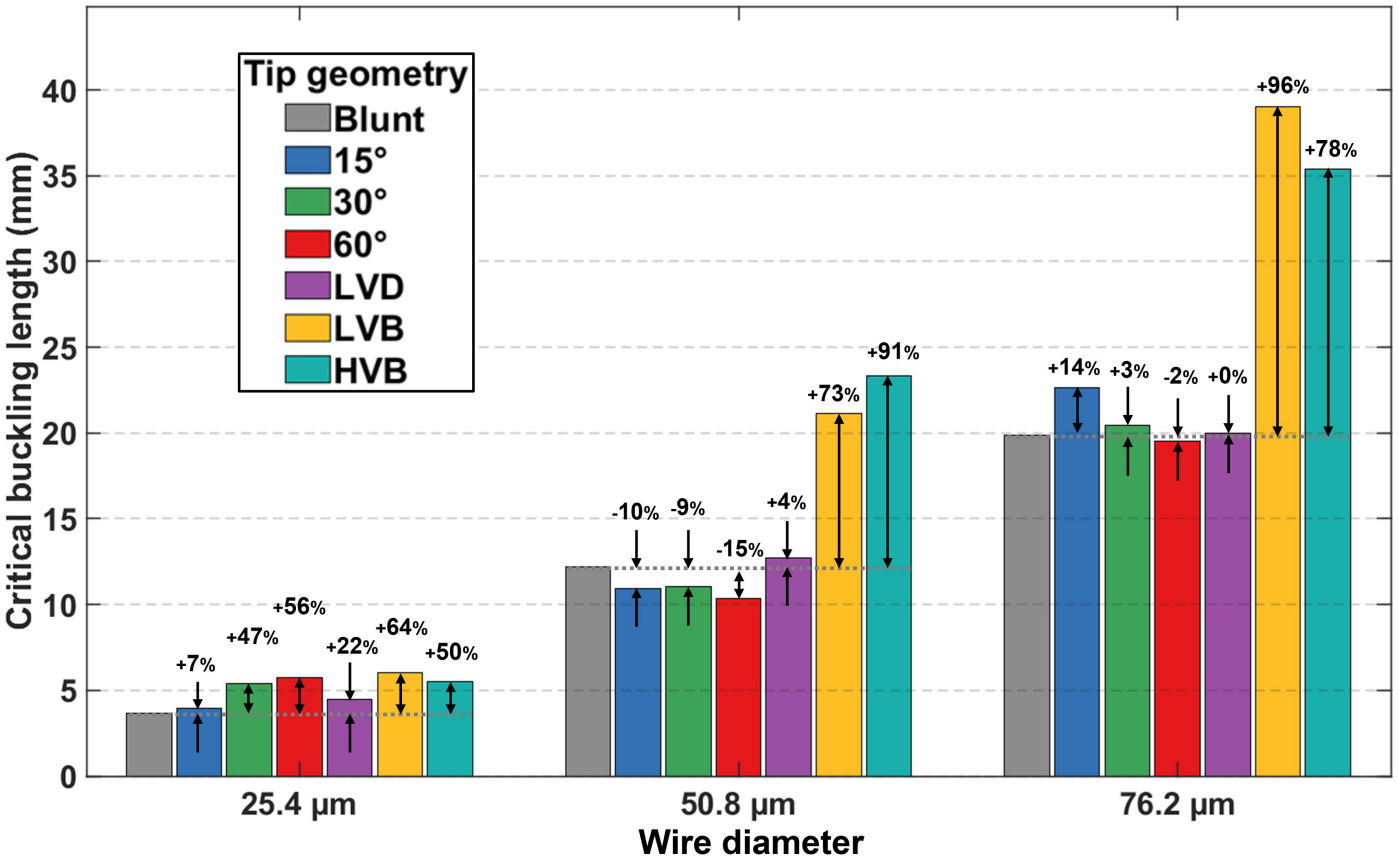
Summary of critical buckling length (statistical threshold value) results from all 21 different wire types. The critical buckling length for each sharp-tip wire type was compared with its blunt-tip counterpart, with the percentage change labeled.

Under each wire type, the correlation between the unsupported length and binary outcome was used to determine the statistical threshold value for the unsupported length, denoted as the critical buckling length, which represented the maximum unsupported length at which a certain type of wire could reliably penetrate the dura and pia mater phantom layers without buckling. The critical buckling lengths for all 21 conditions are plotted in Figure 7, with percentage change marked for each sharp-tip geometry about its blunt tip counterpart as the within-size reference baseline.

As shown in Figure 7, a larger wire diameter led to a longer critical buckling length across all tip shapes. For a circular microwire, the second moment of area scales with the fourth power of diameter, leading to a strong increase in the electrode bending stiffness and thus the critical buckling load with increasing diameter. In contrast, membrane rupture force generally increases linearly with diameter (Obaid et al., 2020b) resulting in overall enhanced buckling resistance with larger-diameter wires. At the same time, both column stability and membrane penetration force are affected by the wire tip geometry. Prior microwire-array studies (Chen et al., 2021; Obaid et al., 2020a) have also reported that the reduction in penetration force achieved by sharpening can depend on wire diameter, which provides additional context for the observed interaction between size and tip geometry in Figure 7.

Within each wire size group, tip sharpening generally increased the critical buckling length. Such buckling enhancement was observed for all six sharp-tip types at a wire size of 25.4 μm. For the 50.8 μm and 76.2 μm-sized wires, the two sharpest tips (LVB and HVB) showed significant critical buckling length improvements, while the single-beveled tips and the LVD tip resulted in very inconsistent improvements and even some reduction in the critical buckling length. The reasons behind these observations were further explored by examining the rupture force and the effective buckling length factor in the following sections.

### 3.2 Membrane rupture force results

**Figure 8:**
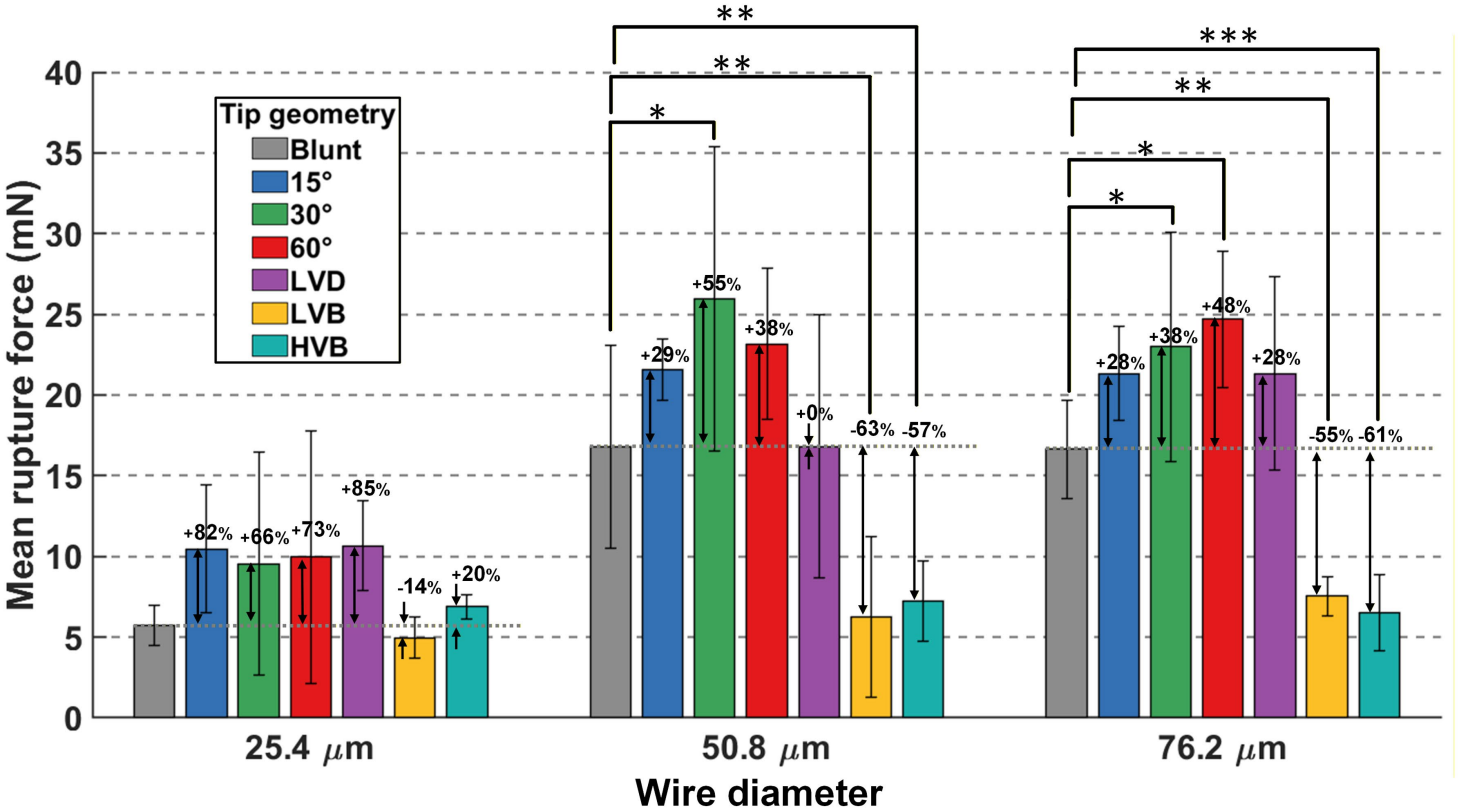
Summary of membrane rupture force results from all 21 different wire types. Mean values are plotted with error bars showing the standard deviation of each wire type. The mean value of each sharp-tip wire performance was compared with its blunt-tip counterpart to calculate the percentage change. Asterisks indicate statistical significance: *p < 0.05, **p < 0.01, ***p < 0.001.

For the 91 successful penetration trials, the recorded membrane rupture force for each wire type is summarized in Figure 8. As shown by the ANOVA results summarized in Table 1, both wire size and tip geometry had a statistically significant impact on the membrane rupture force for successful insertions.

**Table 1:** ANOVA result summary for the effects of wire size and tip geometry on the membrane rupture force.

Parameters	*df*	*F*	*p*
Wire size	2	33.418	< 0.001***
Tip geometry	6	22.359	< 0.001***
Size × tip	12	2.555	0.007**

Bold *p* values indicate statistical significance with *p* < 0.05. Asterisks indicate statistical significance levels: **p < 0.01, ***p < 0.001.

**Figure 9:**
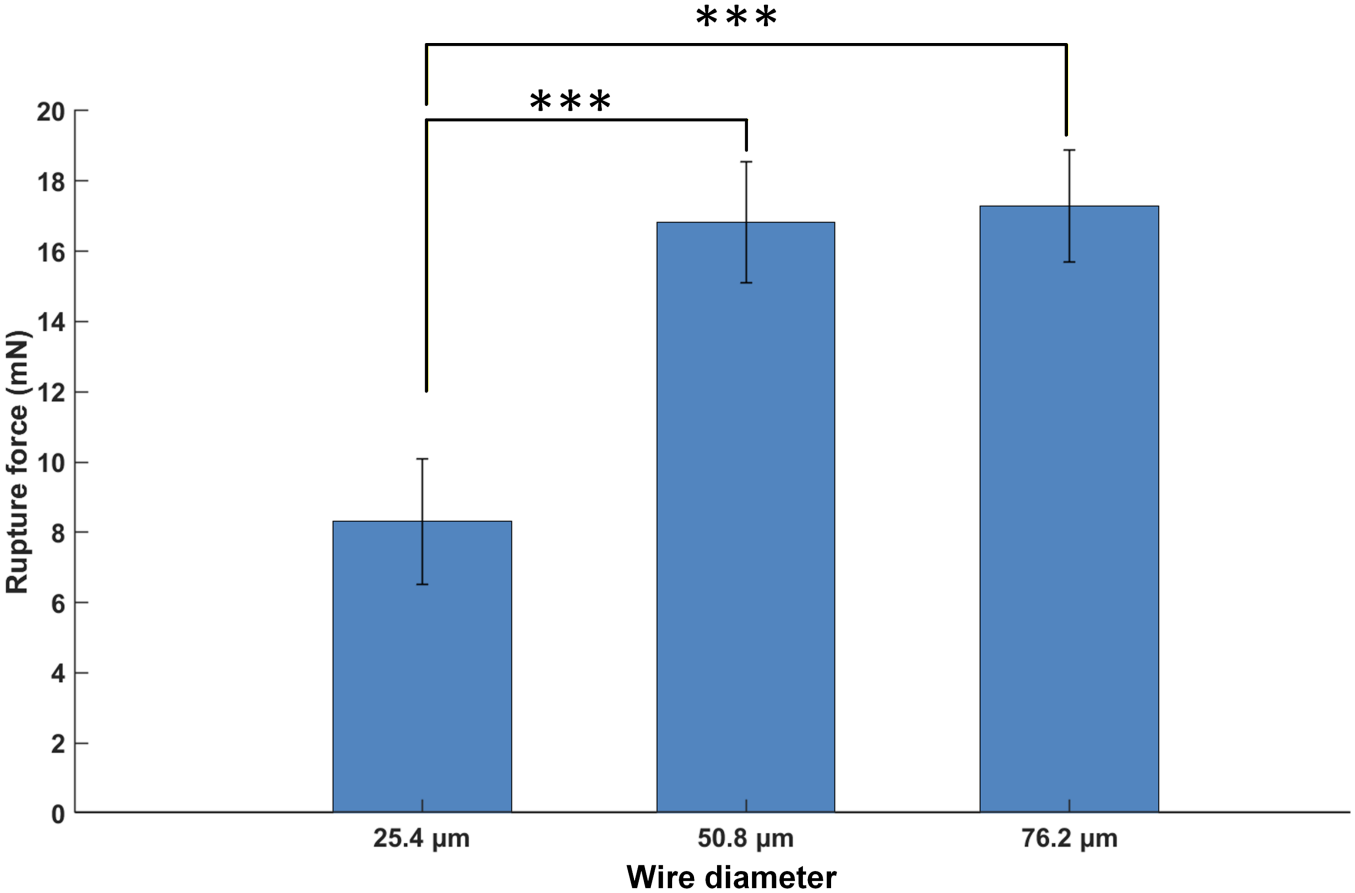
Effect of microwire size on the membrane rupture force. Bars represent the mean rupture force, while error bars show 95% confidence interval. Asterisks indicate statistical significance: *p < 0.05, **p < 0.01, ***p < 0.001.

The effect of wire size on the membrane rupture force is shown in Figure 9. ANOVA showed statistically significant difference between 25.4 μm wires (8.310 ± 0.899 mN) (mean ± standard error, SE) and the larger wires including 50.8 μm wires (16.830 ± 0.899 mN) (mean ± SE) and 76.2 μm wires (17.291 ± 0.800 mN) (mean ± SE), with the 25.4 μm wires generating about half the rupture force as the 50.8 μm ones. Note that the rupture force measurement in this study deviated from the conventional linear trend proportional to the microwire diameter at the 76.2 μm level, likely because rupture force data under each diameter level contained a different number of various tips (as shown in Figure 6), as the main focus of this study was to investigate the critical buckling load and effective length factor.

Among the seven different tip types, compared with blunt-tip microwires, the single beveled-tip microwires all generated statistically significantly larger rupture force (*p* = 0.020 for 15° bevel, *p* = 0.002 for 30° bevel, and *p* = 0.003 for 60° bevel). It could be explained by the fact that the single-bevel tips did not significantly reduce the wire size but the eccentric tip design added uncertainty to the membrane cutting process, as seen by the large standard deviations of the beveled tip conditions, potentially making it harder to achieve dura mater rupture. Conversely, compared with the blunt tip, the sharp HVB and LVB tips yielded statistically significantly lower rupture force (both pairs *p* < 0.001). These conical, sharp tips created a very small tip size, which easily initiated cracks in the dura/pia mimicking layers, thus significantly easing penetration (mean rupture force of HVB and LVB tips at about 50% of that of blunt tips). The LVD tip, with limited sharpness as compared to the blunt tip, generated no statistically significant change in rupture force.

For 25.4 μm wires, despite the mean value variance as marked in Figure 8, no statistically significant difference was found between the blunt tip and any of the six sharp tips. For 50.8 μm wires, with blunt tip as the baseline, pairwise comparisons showed statistically significantly higher rupture force by 30° bevel wires (*p* = 0.013) and significantly lower rupture force by HVB (*p* = 0.002) and LVB (*p* = 0.002) tips. For 76.2 μm wires, 30° bevel (*p* = 0.036) and 60° bevel wires (*p* = 0.020) had significantly higher rupture force, whereas HVB (*p* < 0.001) and LVB (*p* = 0.002) ones showed significantly lower forces. Such observations varied from previous studies, which showed almost equivalent rupture forces between blunt and single-beveled tips. Three facts might have potentially contributed to the different observation (Obaid et al., 2020a). First, compared with previous studies investigating the rupture force under controlled unsupported length and insertion configuration, this study was designed to experimentally explore the borderline critical buckling length value. Thus, more trials were conducted at unsupported lengths that buckled either occasionally or almost always. Such insertion instability, especially for asymmetric single-beveled profiles, might cause rupture to occur with slightly bent wires and/or uneven stress distributions, leading to increased membrane stretching before fracture. Second, the developed phantom material, while calibrated and validated with our *in vivo* measurement dataset, might not fully replicate the membrane rupture mechanism under all conditions. Third, the large variance in insertion trials with single-beveled tips might add uncertainty to the obtained rupture force results.

### 3.3 Experiment-based effective length factor results

**Figure 10:**
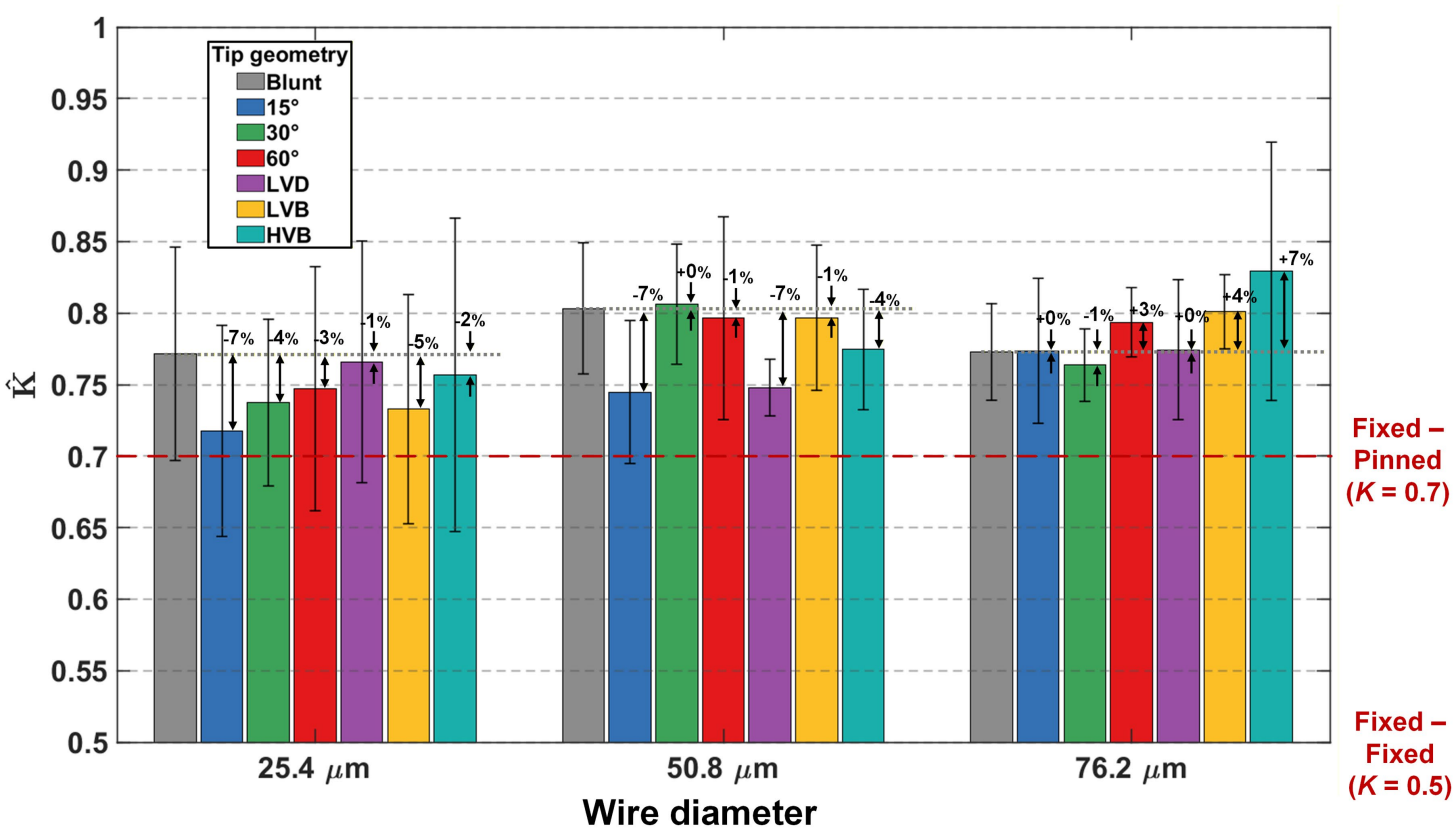
Summary of calculated effective length factor *Kˆ* results from all 21 different wire types. Mean values are plotted with error bars showing the standard deviation of each wire type. The mean value of each sharp-tip wire performance was compared with its blunt-tip counterpart to calculate the percentage change. Asterisks indicate statistical significance: *p < 0.05, **p < 0.01, ***p < 0.001.

The experimental measurement-based effective length factor (*Kˆ*) calculated from all 146 buckled trials is summarized in Figure 10. Note that, across all 21 wire types, the mean *Kˆ* values were higher than the theoretical fixed-pinned boundary condition assumption commonly used (*K* = 0.7). Tip geometry did not exhibit a statistically significant main effect on the effective length factor. However, localized differences observed for certain size–tip combinations suggest that tip geometry may influence anchoring behavior under specific conditions. Accordingly, these observations should be interpreted as condition-specific rather than universal.

**Figure 11:**
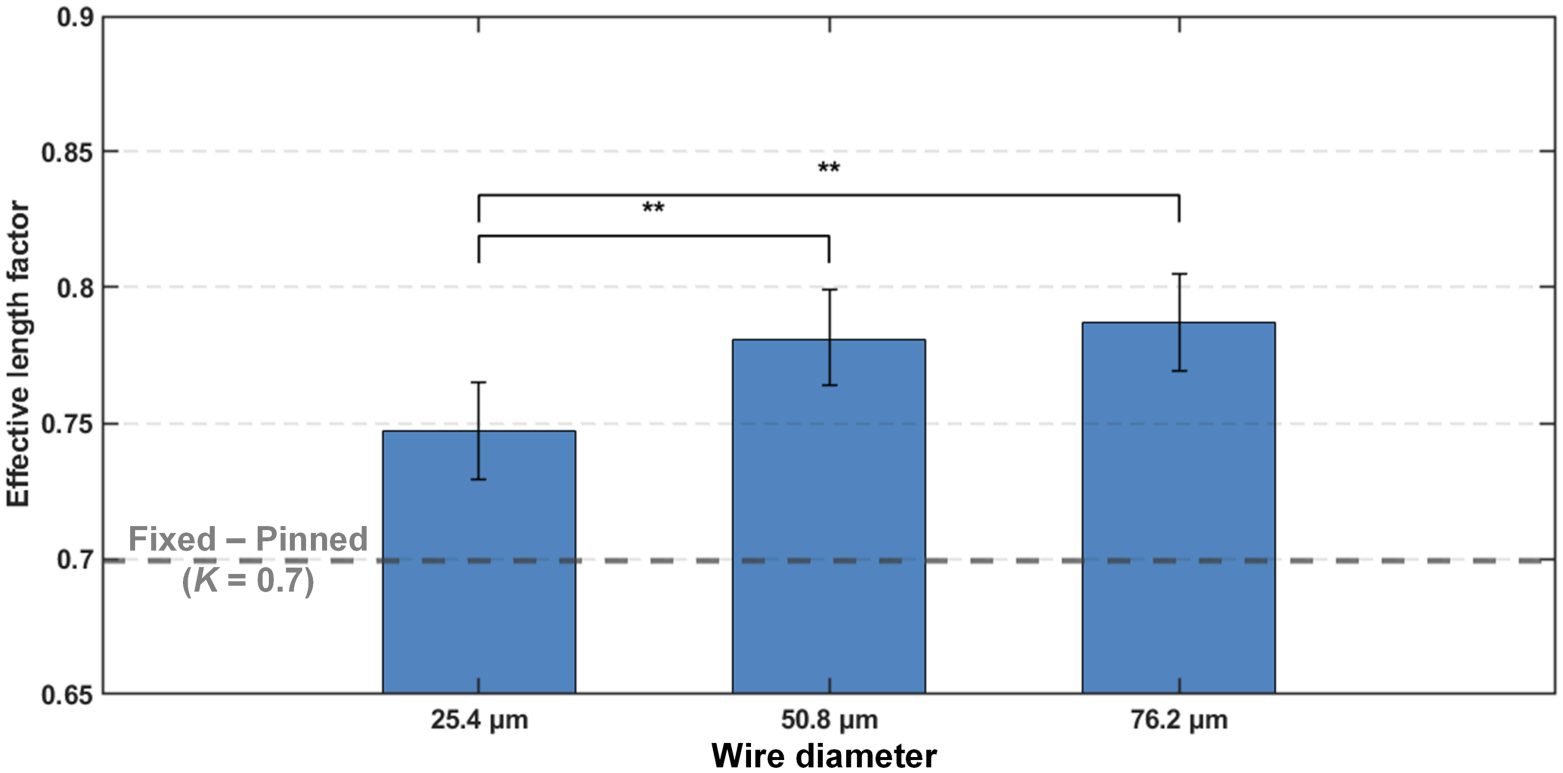
Mean effective length factor (*Kˆ*) for each wire diameter with 95% confidence intervals as error bars. Asterisks indicate statistical significance: *p < 0.05, **p < 0.01, ***p < 0.001.

The effects of wire size and tip geometry on the effective length factor are shown in Table 2. ANOVA revealed that wire size had a statistically significant impact (*p* < 0.001) on the effective length factor. As shown in Figure 11, 25.4 μm wires yielded statistically significantly lower effective length factor (0.747 ± 0.009) (mean ± SE) than the larger 50.8 μm wires (0.781 ± 0.009, *p* = 0.007) (mean ± SE) and 76.2 μm wires (0.787 ± 0.009, *p* = 0.002) (mean ± SE). It indicated that a smaller-sized microwire had better stability against the brain membranes, generating a boundary condition closer to the pinned end at the wire-membrane interface.

**Table 2:** ANOVA result summary for the effects of wire size and tip geometry on the effective length factor (*Kˆ*).

Parameters	*df*	*F*	*p*
Wire size	2	5.659	0.004**
Tip geometry	6	1.003	0.427
Size × Tip	12	0.926	0.524

Bold *p* values indicate statistical significance with *p* < 0.05. Asterisks indicate statistical significance levels: **p < 0.01, ***p < 0.001.

**Figure 12:**
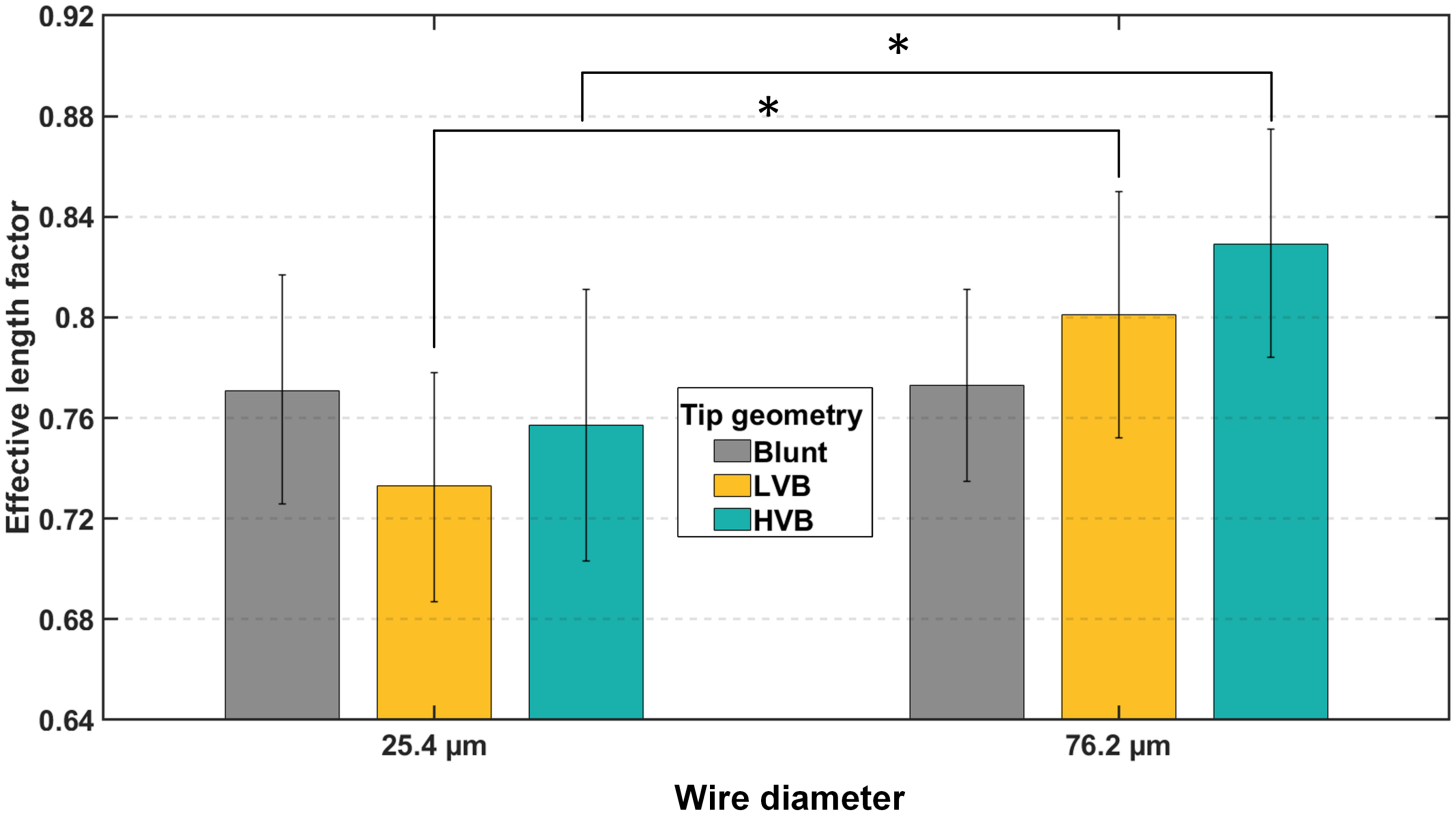
Effect of wire sizes on effective length factor (*Kˆ*) under HVB and LVB tips. The bar chart shows mean *Kˆ* values, and error bars show 95% confidence intervals. Asterisks indicate statistical significance: *p < 0.05, **p < 0.01, ***p < 0.001.

While the tip geometry and wire size do not significantly affect the effective length factor (as in Table 2), pairwise comparisons revealed some interesting outcomes. As shown in Figure 12, under the HVB tip, 25.4 μm wires showed a *Kˆ* of 0.757 ± 0.027 (mean ± SE), significantly (*p* = 0.044) smaller than that of 76.2 μm wires at 0.829 ± 0.023 (mean ± SE). Under LVB tip, 25.4 μm wires showed a *Kˆ* of 0.733 ± 0.023 (mean ± SE), significantly (*p* = 0.046) smaller than that of 76.2 μm wires at 0.801 ± 0.025 (mean ± SE). As a comparison baseline, the two wire sizes with blunt tips showed almost identical effective length factor (0.771 ± 0.023 for 25.4 μm wires and 0.773 ± 0.019 for 76.2 μm wires, mean ± SE). Such results showed that the same conical sharp-tip resulted in reversed impact on the effective length factor between small and large wire sizes. For small wires, such as the 25.4 μm ones, the conical sharp-tip provided greater constraint at the wire-membrane interface, thereby improving wire stability. On the other hand, for thick wires like 76.2 μm, compared to blunt counterparts, the conical sharp-tip worsened the end condition, making the boundary shift toward a freer end. Such a destabilization phenomenon might be explained by the additional instability introduced by the longer tapered section, potential sharp-tip eccentricity-induced axial disruptions, and higher dimpling magnitude introduced by thicker wires and their interaction with the conical sharp-tip.

## 4 Discussion

Combining the critical buckling length, membrane rupture force, and effective length factor results, it could be seen that different wire sizes and tip geometries impact the wire’s buckling resistance through different mechanisms.

For small-sized wires, such as 25.4 μm tungsten wires, which are likely applicable to other smaller cellular-scale metal wires and carbon fiber electrodes, their small size improved wire-membrane interface stability and yielded a small effective length factor, bringing it closer to the pinned-condition *K* value of 0.7. Sharpening the wire could lead to better anchoring of the tip and further reduce the effective unsupported length against buckling. On the other hand, the benefits of wire sharpening on rupture force reduction might be limited, especially for the asymmetrical tip geometries, including single-beveled and LVD tips, which introduced higher variance than blunt tip baselines. As a result, the choice of a sharp-tip for thin microwires should primarily focus on stabilizing the wire anchoring point and reducing the rupture force variance introduced by asymmetrical tip geometries.

For large-sized wires, such as the 50.8 and 76.2 μm tungsten wires in this study, a single-beveled tip might provide a minor benefit in improved end condition; however, this benefit was offset by the significantly increased membrane rupture force and the high variance of such insertions. Large-tip angle conical tips, like the LVD one, had limited change for large-sized ones, as they function similarly to blunt tips across the large footprint. Sharp conical tips, such as the HVB and LVB tips with long tapers and sharp-tip angles, improved buckling resistance mainly through a significant reduction (over 50%) of the membrane rupture force. While a sharper tip might reduce rupture force, it should be noted that the sharp-tip itself could also introduce instability and worsen the tip-membrane boundary, leading to reduced wire critical buckling length. Thus, the choice of conical tip sharpness, especially for larger diameter wires, depends on the compromise between rupture force reduction and wire end stability.

For all wire sizes and tip geometries investigated in this study, it was found that the conventional fixed-pinned end condition assumption (K = 0.7) for critical buckling load calculation via Euler’s column buckling equation tended to be questionable and might lead to overestimation of the allowable critical buckling length (thus the maximum insertion depth possible). Based on this study and the experiment measurement-based calculation of the effective length factor, it would be within the 0.72–0.82 range. The experimentally observed range suggests that adopting K ≈ 0.8 may be a conservative design choice for membrane penetration scenarios, helping to avoid overestimation of allowable unsupported length. From a design perspective, selecting a value near the upper bound of the experimentally observed range provides a conservative estimate that mitigates the risk of structural instability. But the range observed in this study also had its limitations. In practical neural interface applications, microwires are sometimes inserted in bundled configurations rather than individually. Such arrangements may alter buckling behavior through increased collective stiffness, lateral constraint between neighboring wires, and modified boundary conditions at the membrane interface. While the present study focuses on single-wire mechanics to isolate fundamental penetration behavior, extending this framework to bundled insertions represents an important direction for future work. Furthermore, the experimentally observed effective length factor range (0.72–0.82) was obtained under controlled insertion at a rate of 100 μm/s. Further investigations at different insertion speeds would be needed to identify the impact of insertion speed on the effective length factor and thus the critical buckling load.

The findings of this study are broadly consistent with prior investigations into neural electrode insertion mechanics (Dong et al., 2020; Marshall et al., 2016). Previous work has demonstrated that increasing electrode diameter enhances bending stiffness and reduces the buckling chance, while tip geometry plays a critical role in modulating membrane rupture force (Dong et al., 2020; Chen et al., 2021; Thielen and Meng, 2021; McNamara et al., 2024). The present results reinforce these observations regarding the chances of rupture force and, more importantly, experimentally illustrate the impact of microwire configuration on the effective length factor and critical buckling load. Notably, the effective length factors measured were above the classical fixed–pinned assumption (*K* = 0.7) often adopted in electrode design. These new findings revealed the importance of experimentally informed estimates for predicting unsupported length for successful membrane penetration. This perspective complements prior insertion studies by emphasizing boundary-condition sensitivity as an additional design consideration, alongside conventional diameter choice and tip sharpening, to minimize membrane rupture force.

## 5 Conclusion

An experimental investigation of microwire-based brain electrode buckling was conducted in this study using microwires with various sizes and tip geometries. The microwire’s buckling resistance was quantitatively evaluated via the critical buckling length, membrane rupture force, and the actual effective length factor as used in Euler’s column buckling equation. The following conclusions might be drawn based on the study:

- Conical tips with sharp point angle dramatically reduce the membrane rupture force as compared to blunt ones, and they outperform single-beveled tips and short-taper conical tips in terms of generating repeatable membrane rupture with less variance.
- Smaller microwires tend to anchor better against the brain membrane, generating a more stable wire-membrane interface closer to the ideal pin end condition.
- Tip sharpening choices for small microwires should focus on optimizing the tip anchoring mechanism and minimizing rupture force uncertainty introduced by tip asymmetry.
- For thick microwires, sharp conical tips bring the most enhancement to critical buckling length mainly due to significantly reduced membrane rupture force. But compromises also need to be made in tip sharpness, as further-sharpened tip geometries with a smaller taper ratio increase the effective length factor and thus negatively impact the critical buckling load.
- For the theoretical prediction of a microwire electrode’s critical buckling load based on Euler’s buckling equation, based on the experiments in this study conducted at 100 μm/s insertion speed, it would be recommended to use an effective length factor *K* of about 0.8 to provide a more conservative estimation of the real wire-membrane interface condition.

## Data Availability

The raw data supporting the conclusions of this article will be made available by the authors, without undue reservation.
